# Magnetic Fields and Reactive Oxygen Species

**DOI:** 10.3390/ijms18102175

**Published:** 2017-10-18

**Authors:** Huizhen Wang, Xin Zhang

**Affiliations:** 1 High Magnetic Field Laboratory, Chinese Academy of Sciences, Hefei 230031, China; huizhenwang@hmfl.ac.cn; 2 School of Life Sciences, University of Science and Technology of China, Hefei 230027, China; 3 Key Laboratory of High Magnetic Field and Ion Beam Physical Biology, Hefei Institutes of Physical Science, Chinese Academy of Sciences, Hefei 230031, China

**Keywords:** reactive oxygen species (ROS), magnetic field (MF), static magnetic field (SMF), extremely low frequency electromagnetic field (ELF-EMF), radio frequency electromagnetic radiation (RF-EMR)

## Abstract

Reactive oxygen species (ROS) ubiquitously exist in mammalian cells to participate in various cellular signaling pathways. The intracellular ROS levels are dependent on the dynamic balance between ROS generation and elimination. In this review, we summarize reported studies about the influences of magnetic fields (MFs) on ROS levels. Although in most cases, MFs increased ROS levels in human, mouse, rat cells, and tissues, there are also studies showing that ROS levels were decreased or not affected by MFs. Multiple factors could cause these discrepancies, including but not limited to MF type/intensity/frequency, exposure time and assay time-point, as well as different biological samples examined. It will be necessary to investigate the influences of different MFs on ROS in various biological samples systematically and mechanistically, which will be helpful for people to get a more complete understanding about MF-induced biological effects. In addition, reviewing the roles of MFs in ROS modulation may open up new scenarios of MF application, which could be further and more widely adopted into clinical applications, particularly in diseases that ROS have documented pathophysiological roles.

## 1. Introduction

Reactive oxygen species (ROS) are a series of highly active radicals, irons and molecules that have a single unpaired electron in their outer shell, including free oxygen radicals, such as superoxide anion (•O_2_^−^), hydroxyl radical (•OH), and single oxygen (^1^O_2_), and non-radical ROS, for instance, hydrogen peroxide (H_2_O_2_), organic hydroperoxides (ROOH), and hypochloric acid (HOCl) [1]. It is widely accepted that ROS at low levels can act as second messengers and activate signaling cascades in response to gene expression, cell proliferation, apoptosis, and other intracellular pathways [2]. On the other hand, excessive ROS could attack membrane phospholipids, impair mitochondrial function, and damage proteins, lipids, DNA, RNA, and sugar to disrupt normal cellular processes [3].

## 2. Reactive Oxygen Species Generation and Elimination

### 2.1. ROS Generation

Previous studies revealed that ROS are produced both by enzymatic and non-enzymatic processes. The electron transport chain (ETC) in the cell respiration process at mitochondrial membrane is the main source of ROS production. It is well documented that mitochondria generate ATP by oxidizing glucose/lipids/amino acids and transferring electrons to the ETC, which ultimately delivers them to O_2_. During ATP synthesis, electrons may escape from the ETC, especially from complexes I and III, and react with O_2_ to form •O_2_^−^ [4]. In case of mitochondrial dysfunction, ATP production collapses and mitochondria produce large quantities of ROS [5]. Because •O_2_^−^ is constantly produced during cell respiration, and it can be catalyzed to H_2_O_2_ and other ROS either in the mitochondrial matrix (by manganese dependent superoxide dismutase (SOD), MnSOD), or cytosol (by copper and zinc dependent SOD, Cu/ZnSOD), mitochondria are considered to be the major sources of intracellular ROS [6]. Besides mitochondria, peroxisome is another major organelle of cellular ROS generation because •O_2_^−^ and H_2_O_2_ can be generated through xanthine oxidase in the matrix or membranes of peroxisomes [7,8]. Additionally, ROS could also be produced by a family of membrane-bound enzymes, such as NAD(P)H oxidases, especially in neutrophils and macrophages cells [9,10]. Asides from these ROS generation processes under normal physiological conditions, activation of oncogenic signals, mitochondrial dysfunction, and aberrant metabolism are all intrinsic factors that could contribute to high ROS levels in cancer cells [11].

### 2.2. ROS Elimination

Large amount of evidences illustrated that detoxification from ROS is facilitated by non-enzymatic molecules, including glutathione (GSH), vitamin A, C, and E, or through antioxidant enzymes that primarily scavenge different types of ROS [12]. Most living organisms evolved the antioxidant defense system to protect cells from excessive ROS, which includes SOD, catalase (CAT), glutathione peroxidase (GSH-Px), glutathione reductase (GSH-R), and other non-enzyme molecules. Among these antioxidant enzymes, SOD is primarily important for ROS detoxification. The glutathione system consists of GSH, GSH-Px, GSH-R, and glutathione S-transferase (GST). GSH can protect cells from oxidative stress through reducing disulfide bonds of cytoplasmic proteins, and itself is oxidized to glutathione disulfide (GSSG) during the process [12]. Therefore, the activities of SOD, CAT, and GSH-Px, and the ratio of GSH/GSSG could serve as biomarkers for indicating the oxidase stress in cells, or even intracellular ROS levels. Additionally, the levels of lipid peroxidation (LPO) products (malondialdehyde (MDA) and 4-hydroxynonenal (4-HNE)) are another indicator of oxidative damage [13].

## 3. The Types of Magnetic Fields (MFs)

In general, magnetic fields can be generated by permanent magnets or electric currents, and the latter one is usually called electromagnetic fields (EMFs). It is well known that all living organisms are surrounded by various MFs, which are produced by natural or man-made sources. Hence, more and more people begin to pay attention to whether these MFs have harmful or beneficial effects on human beings. In fact, many studies looked at the anti-inflammatory effects of pulsed EMFs in several in vitro [14,15,16], in vivo [17,18,19], and clinical studies [20], particularly in musculoskeletal researches.

MFs can be divided into static MF (SMF) and time-varying/dynamic MF, which can be further classified into different categories depending on multiple parameters, such as frequency, intensity, or application in practice. If the intensity and direction of a MF sustained, it is called “SMF”. Earth MF is between 35 and 70 μT (depending on location), which could be perceived by certain animals for orientation, such as birds, salamanders, and turtle [21]. Man-made SMFs are usually generated by permanent magnets or EMFs in the form of direct current (DC) with no change in intensity or direction over time (frequency of 0 Hz) [22]. Depending on intensity, MFs can be classified into weak (<1 mT), moderate (1 mT–1 T), strong (1–20 T), and ultra-strong (>20 T) MFs.

According to frequency, MFs are consisted of extremely low frequency electromagnetic fields (ELF-EMF, <300 Hz), intermediate frequency (300 Hz–10 MHz), and radiofrequency (10 MHz–300 GHz) [23]. One of the most commonly seen dynamic MFs is generated by alternating current (AC) power line (60 Hz in USA and Canada, 50 Hz in the rest of the world) [22,24]. Mobile phone frequencies are usually 900/1800 MHz, which belong to radiofrequency. Besides these category methods, relying on the MF spatial distribution, there are homogeneous or inhomogeneous MFs. In the current review, we only focus on the effects of SMFs, ELF-EMFs, and RF-EMRs (radio frequency electromagnetic radiations) on ROS levels.

## 4. The Effects of Magnetic Fields on ROS

There are various evidences showing that MFs could affect ROS. However, the reported experimental results are miscellaneous, which is largely due to the different MF parameters, biological samples examined as well as experimental set up [1]. Therefore, we summarize reported studies based on MF types and their effects on ROS levels. Some key details are also listed in this review, such as MF intensity, frequency, exposure time, cell lines, assay time-point, as well as some other experimental details. Although it seems verbose, but we think these experimental details are very critical for people to analyze MF-related experimental results.

### 4.1. Static Magnetic Field (SMF)

Increasing studies have been conducted to investigate whether and how SMFs affect ROS levels [25], but the results are still not fully consistent. While most of the studies found that SMFs increased ROS levels, whereas there are several other reports revealed opposite effects. In addition, a few evidences also indicated that ROS levels were not affected by SMFs. We summarize and compare most reported results of SMFs on ROS, and categorize them into “increase”, “decrease”, and “no change” based on the effects of SMFs on ROS (Table 1).

#### 4.1.1. SMFs That Increase ROS Levels

Most studies so far about the effects of SMFs on ROS showed elevated ROS levels after SMFs exposure. For example, two studies in human neuroblastoma SH-SY5Y cells found that the intercellular ROS could be increased by SMFs of different intensities [27,28]. More specifically, Calabro et al. reported that 2.2 mT SMF exposure for 24 h significantly increased H_2_O_2_ (about 21%) [27]. The same effect was confirmed by Vergallo and his colleagues, that inhomogeneous SMF (31.7–232.0 mT) exposure for 24 h induced •O_2_^−^ elevation (23%) [28]. In 2006, De Nicola et al. reported that exposure of human monocyte tumor cells (U937) to 6 mT SMF for 2 h could trigger intracellular ROS increased [29]. High-gradient magnets (1.2 T, 24 h) were revealed to be able to induce continuous cellular ROS production of human monocytic leukemia cells (THP-1) by Zablotskii et al. [32]. In addition, Zhao et al. found that cellular ROS levels in three cell lines, human-hamster hybrid A(L) cells, mitochondria-deficient rho(0) A(L) cells, and double-strand break (DSB) repair-deficient XRS-5 cells, were significantly increased after 3 h exposure to a 8.5 T strong SMF [33].

Since ROS are indicated to be involved in the vasculogenesis and cardiomyogenesis of mouse embryonic stem (ES) cells [42,43], two studies were performed in mouse ES cells and derived cells. Bekhite et al. found that SMFs induced endogenous ROS increase in embryoid bodies in a dose-dependent manner (10 mT SMF generated a more significant effect than 1 mT SMF) [36]. Their later study found that endogenous ROS were elevated by 0.2–5 mT SMFs dose-dependently in Flk-1^+^ cardiac progenitor cells derived from mouse ES cells [34]. Furthermore, they proposed that ROS were generated through NADPH oxidase because the NOX-4 mRNA was up-regulated upon 1 mT SMF exposure for six days [34].

There are also a few other studies that investigated the influences of SMFs on ROS in other cell types, as well as plants. For example, Bae et al. found that the intracellular ROS were significantly increased in mouse normal liver cell line (NCTC 1469) under exposure of 0.4 T for 1/24/48/72 h [35].

#### 4.1.2. Differential Effects of SMFs on ROS Levels

Although most studies found that SMFs elevated cellular ROS levels, there are also a few evidences reported different results. For example, intermittent SMF (induction level of 370 mT, on/off cycles of 1 h/day, for four consecutive days) exposure did not affect intracellular ROS level in human foetal lung fibroblasts (MRC-5) [40]. Since direct ex vivo measurements of ROS were difficult not only because of limited amount of cochlea specimens, but also the extremely short lifetime of ROS, so LPO levels could be used as indicators for oxidative stress. Some studies applied LPO products (MDA or 4-HNE) as markers of ROS level. Politanski et al. found no significant difference of cochlear ROS levels in male C57BL/6 mice between groups exposed to 5 mT SMF for each time-point (1, 3, 5, 7, 14 days) and controls, the activities of SOD/CAT enzymes were significantly elevated after exposure for three days, but not for other exposure time [44]. Cisllag et al. found that exposure of human bronchial epithelial cells (A549) to inhomogeneous SMFs of 389 mT, either at lower or upper position, for 30 min, did not alter the levels of intrinsic ROS in PBS-treated cells, but could significantly decrease ROS level induced by ragweed pollen extract (RWPE) [38]. In addition, studies conducted in prokaryota (*E. coli* and *S. aureus*) observed no significant difference of ROS level after 100 mT exposure for 4 h [41].

The discrepancy about the different effects of SMFs on ROS levels could be resulted from the differences in cell types, MF intensity, poles or direction, exposure time, or even assay time-point after exposure. Intriguingly, as opposite to the externally applied MFs in most studies, a low intensity MF-hypomagnetic field (HMF, 0.2–2 μT), created by shielding geomagnetic field (GMF, 45–60 μT), could induce a H_2_O_2_ decrease after exposure for 24 h in human fibrosarcoma cancer cell line (HT1080) and bovine pulmonary artery endothelial cells (PAEC), but not in human pancreatic cancer cell line (AsPC-1), as compared to unexposed cells in GMF, speculating that ROS level might be elevated by increased MF intensity in a cell type-specific manner [26]. Contrary to the study, HMF exposure for three days could trigger ROS increased significantly in mouse primary skeletal muscle cell, as compared to cells in the GMF condition, indicating that ROS level might be decreased along with MF intensity going up [39]. Moreover, Sullivan et al. found a statistically significant increase in ROS level in human diploid embryonic lung fibroblast cells (WI-38) after exposure to SMF (230–250 mT) during the first 18 h after seeding, but not after continuously 5-days of exposure [31]. They also showed that cell lines with a lower growing rate tended to be less affected, which was probably because of their inherent physiology variations [31]. In addition, Poniedzialek et al. also showed that in human peripheral blood neutrophils, ROS level was significantly reduced after 60 mT SMF exposed for 15 min, but recovered to control level at 30 min. More surprisingly, ROS level was later visibly elevated at 45 min, but only in the cells that were exposed to the south poles of the magnets, not north poles [30], indicating that the effects of SMFs on cellular ROS were dependent on both exposure time and the magnetic poles. It could be argued that these effects are likely due to MF direction, rather than the magnetic poles themselves.

In summary, multiple experimental factors all contribute to the differential effects of SMFs on ROS levels in independent reports. In fact, a previous study has already showed that ROS level was critically dependent on cell density. More specifically, cells plated at lower cell density had a higher ROS level than cells plated at higher cell density [45]. Therefore, it is not surprising that ROS could be differentially regulated in different cell conditions. Researchers should do more systematical analysis for the different MF intensity, exposure time, and cell types to get a more complete understanding about the effects of SMFs on ROS.

### 4.2. Extremely Low Frequency Magnetic Field (ELF-EMF)

Large bodies of literatures have been surveyed with an emphasis on whether ELF-EMFs affect ROS at in vitro and in vivo levels, which was probably due to the prevalent exposure of human bodies to ELF-EMFs that was generated by power lines. However, similar to SMFs, these studies about ELF-EMFs produced seemingly inconsistent results as well, which is reasonable because they have more variable parameters than SMFs. In Table 2, Table 3 and Table 4, as well as the following section, we group them based on ROS changes, such as ROS increase/decrease (Table 2 and Table 4), no change (Table 3 and Table 4), as well as in humans (Table 2 and Table 3) or in mice and rats (Table 4). In each group, we further analyze them based on cell types, because we previously found that cell type is a key factor that determines the MF-induced cellular effects [46,47].

#### 4.2.1. ELF-EMFs That Increase ROS Levels

##### Human Leukemia Cell Line (K562), Human Neuroblastoma Cells (SH-SY5Y), Human Amniotic Epithelial Cells (FL) and Other Human Cells

Considering that increased ROS were observed in several hematopoietic malignancies, including acute and chronic myeloid leukemias, K562 was frequently used to examine the effects of ELF-EMFs on intracellular ROS levels. In fact, there are four sequential studies all found that •O_2_^−^ level was increased in K562 after ELF-EMFs exposure (50 Hz 0.025–5 mT for 1–24 h) [49,50,51,52].

Similar to K562, SH-SY5Y was also preferentially used to explore the influences of ELF-EMFs on ROS. For example, one study verified that exposure of SH-SY5Y to 50 Hz 1 mT ELF-EMF for 24/48/72 h significantly increased ROS level [55]. Previously, in 2014, Luukkonen et al. found that 100 Hz 100 μT ELF-EMF exposure for 24 h induced delayed effects in SH-SY5Y cells (ROS were visibly increased at 15 days after exposure, but not at eight days after exposure) [53], indicating that assay time-point after exposure is important.

**Figure 1:**
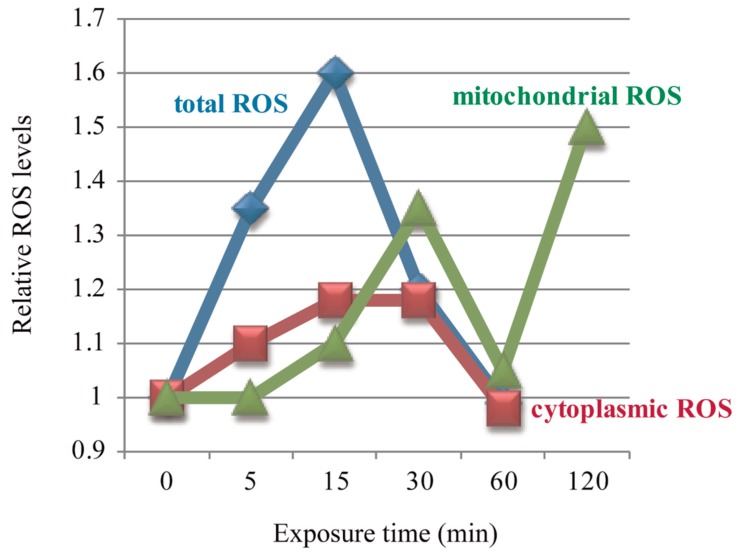
Electromagnetic fields (EMFs)-induced ROS level changes were time-dependent. Figure was made based on results from References [56,57,58].

Multiple studies have shown that ELF-EMFs could induce ROS changes very rapidly [56,77,78], and in a time-dependent manner [56,57,58]. Feng and his colleagues reported three studies about the effects of ELF-EMFs on intracellular ROS levels in human FL cells. They found that cellular ROS were elevated after 50 Hz 0.4 mT ELF-EMF exposure for 5/15/30 min, with a peak at 15 min, and then returned to control level at 60 min when compared to control cells [56,58] (Figure 1) and the intensity threshold that could trigger the effect of ELF-EMFs on ROS was 0.1–0.2 mT [56]. The change pattern of cytoplasmic •O_2_^−^ was exactly the same as total ROS, while mitochondrial ROS were not increased until after 15/30 min exposure, but also returned back to control level at 60 min [56], which was in accordance with their previous result that mitochondrial ROS were increased after 0.4 mT EMF exposure for 30 min, then returned to normal level at 60 min, but elevated again after 120 min exposure [57]. These three studies revealed that the effects of ELF-EMFs on cellular ROS were both field intensity- and exposure time-dependent. In the meantime, the mitochondrial permeability transition (MPT) was detected to be increased after exposure for 60 min, but the mitochondrial membrane potential (Δψm) showed no alteration [58]. Feng et al. observed that 0.4 mT 50 Hz ELF-EMF exposure for 15 min could activate the epidermal growth factor receptor (EGFR) clustering in FL cells, which could be completely reversed by the NADPH oxidase inhibitor, DPI [56], which indicates that the 50 Hz EMF-induced EGFR clustering was mediated by ROS. More studies about the ELF-EMFs effects on ROS are summarized in Table 2.

##### Mice and Rat Studies at Cellular and Animal Levels

Besides human cells, there are numerous studies conducted on mice or rat at both cellular and animal levels to examine whether ELF-EMFs affect ROS levels (Table 4). Most studies in mouse cell lines and mice models showed an increased ROS level after exposure. For example, cellular ROS were significantly increased in mouse embryonic stem (ES) cell-derived embryoid bodies (EBs) after exposure to either 1 or 10 mT EMFs (AC or DC) for 7 days, 8 h/day, in a dose-dependent manner (~2-fold in 1 mT group, ~6-fold in 10 mT group) [22]. In mouse bone marrow-derived (MBM) macrophages, the 50 Hz 1 mT ELF-EMF exposure increased intracellular ROS significantly (1.4-fold) after 45 min exposure, but not at 5/15/30 min [23]. In fact, this time-dependency was also supported by other independent reports. For example, Chen et al. found that in mouse embryonic fibroblasts (MEF), 50 Hz 2 mT ELF-EMF increased ROS after 2/6 h exposure, but returned to normal level after 12/24 h [79]. A significant ROS elevation was induced by ELF-EMF (7.5 Hz, 0.4 T) exposure for 2 h in primary mouse T cells and human T-leukaemia (Jurkat) cells, but the change was transient and modest, and returned to normal level after 3 h [48], which was consistent with a previous study performed in Jurkat cells that ROS level was not increased following ELF-EMF (50 Hz) exposure of 1 h intermittent (5 min on/10 min off) [73]. These independent studies all indicated that intracellular ROS levels might fluctuate during MFs exposure.

Similar to mouse cell lines, ELF-EMFs could also induce ROS elevation in rat cells [80,82], or rodent tissues in time-dependent manner [84]. Morabito et al. reported that 50 Hz 1 mT (but not 0.1 mT) ELF-EMF exposure for 30 min could induce ROS elevation in undifferentiated (but not differentiated) rat pheochromocytoma PC12 cells [80]. However, de Groot et al. did not observe any significant ROS changes in either naive or chemically stressed PC12 cells after 50 Hz 1 mT ELF-EMF exposure for 30 min or 48 h [88]. In contrast, 0.1 mT 60 Hz ELF-EMF exposure for five days could elicit ROS level increase in rat peritoneal neutrophils [82], which is lower than the previously proposed intensity threshold of 0.1–0.2 mT [56,77]. Goraca and his colleagues exposed male Wistar rats to 40 Hz 7 mT ELF-EMF for two weeks and detected dramatic ROS elevation in rat heart tissue after exposure for 60 min/day, but not for 30 min/day [84].

#### 4.2.2. Differential Effects of ELF-EMFs on ROS Levels

Most reports found that ELF-EMFs could increase ROS levels, but there are also evidences indicated different results [67,68,72,73,74,75,86,88] (Table 2, Table 3 and Table 4). For example, it was shown that low intensity ELF-EMFs could reduce ROS levels in human keratinocytes, dermal fibroblasts [72] and neutrophils [76]. In addition, ELF-EMFs could also decrease H_2_O_2_, cisplatin, oxygen-glucose deprivation (OGD), and hypoxia or hypoxia/reoxygenation (H/R)-induced ROS elevation in several cell lines [68,69,70,71,78,86] (Table 2).

The exact effects of ELF-EMFs on ROS levels in vitro and in vivo are dependent on multiple factors, including but not limited to the MF intensity/frequency/exposure time, cell lines, or tissues of animal models. Any parameter changes could potentially cause variable experimental results. For example, Calcabrini and his colleagues observed that 0.05/0.1 mT 50 Hz exposure for 1/2 h could increase ROS level in human keratinocyte cell line (NCTC 2544), but return to normal level at 4 h, while a lower intensity (0.025 mT) or a higher intensity (0.15/0.2 mT) did not cause ROS changes [59]. In addition, Manikonda showed that EMF-induced ROS level changes in rat brain are tissue- and intensity-specific [85]. Moreover, ELF-EMFs frequency is also important [66,67]. Therefore, all detailed information should be carefully recorded for EMF-related studies.

### 4.3. Radio Frequency Electromagnetic Radiation (RF-EMR)

The potential harmful effects of RF-EMRs on human health have been concerned over decades, and many researches attempted to evaluate whether RF-EMRs could elevate intracellular ROS levels. Unlike the lower frequency EMFs or SMFs, RF-EMRs of over 900 MHz could transfer energy and exert thermal effects on biological matters [90]. However, similar to the lower frequency EMFs and SMFs, RF-EMRs studies on ROS levels also produced variable results (Table 5).

#### 4.3.1. RF-EMRs That Increase ROS Levels

Notably, the association of RF-EMRs with ROS increments was consistent in both human ejaculated semen and male Wistar rat sperms [91,92,99,100]. Agarwal and his colleagues reported that ROS level was significantly increased in human ejaculated semen after 850 MHz RF-EMR exposure for 1 h [91], which further confirmed by De Iuliis et al. that the total ROS of human spermatozoa was also significantly elevated after 1.8 GHz radiation for 16 h in a SAR (specific absorption rate)-dependent manner [92]. Similar results were detected in sperm of male Wistar rats. Both 900 MHz for 35 days, 2 h/day and 10 GHz for 45 days, 2 h/day could induce a significant increase in ROS [99,100]. These four reports implied that RF-EMRs emitted from cell phones might have a significant effect on the reproductive system of man and male rats, thereby resulting in male infertility, indicating the potential threaten of EMRs to human health. These findings were further confirmed in drosophila bodies or ovaries of females (shown in Table 5). Exposure of male/female bodies for 6/24/96 h significantly increased ROS, whereas 0.5/1 h did not, indicating of exposure time dependency, while ROS levels in ovaries were significantly increased after radiation for 0.5/1 h or 6/24/96 h [102]. There were studies found that the generation pattern of RF-EMRs [98] or SAR [96] could contribute to the differential effects of RF-EMRs on ROS. Friedman and his colleagues showed that ROS increased by 875 MHz radiation was mediated by membrane-associated NADH oxidase in HeLa cells and Rat1 (rat immortalized but not transformed fibroblasts) [109].

#### 4.3.2. RF-EMRs That Have No Effects on ROS Levels

There are also some studies showing that RF-EMRs did not affect ROS in human, mouse, or rat cells, or Caenorhabditis elegans (*C. elegans*). For example, Luukkonen et al. found that no significant ROS change in SH-SY5Y after 872 MHz radiation for 1 h [93,103]. Some previously discussed studies also found that RF-EMRs did not affect ROS levels in certain experimental conditions [93,96,98,102]. More detailed information was shown in Table 5. Similar to the effects of SMFs and ELF-EMFs on ROS levels, the influences of RF-EMRs on ROS are still inconclusive because they are also cell-type-, frequency- and exposure time-dependent.

## 5. Underlying the ROS Changes Induced by MFs

As we have discussed, cell types, MF intensity/frequency/exposure time, tissues of animal models, assay time-point after exposure, cell plating density, or other experimental details all contribute to the differential effects of MFs on ROS levels in independent studies. In addition, for dynamic MFs, more parameters are involved. For example, although intermediate frequency EMFs (300 Hz–10 MHz) are barely studied, one study showed that 500 Hz EMF exposure for just 1 min could trigger a 1.9-fold ROS increase in two different types of cells, but it only happened at 500 Hz 20 V/m, not 10 V/m EMF [110].

Although enormous evidences showed that MFs affect ROS levels, there is no consensus about their exact effects. Besides the different parameters mentioned above, this is also partially due to the reason that the underlying mechanisms of MF-induced ROS changes still remain elusive. Although it is well acknowledged that ROS level is dependent on the dynamic balance between ROS generation and elimination, only a few studies explored whether the production or elimination process of ROS was affected by MFs. The reported influences of MFs on the activities of the antioxidant enzymes are summarized in Table 4. For example, Shine et al. showed that 150–200 mT SMF could enhance ROS production (•O_2_^−^, •OH and H_2_O_2_) intensity-dependently in embryos and hypocotyl after only 1 h of exposure to soybean seeds, while SOD activity was reduced in the hypocotyl of soybean seedlings [37] (Table 6). In contrast, exposure of broad bean (*Vicia faba* L.) to 15 mT SMF (8 h/day for 8 days) increased SOD activity, but decreased CAT, suggesting that the antioxidant defense system was suppressed and potentially caused ROS accumulation [111]. These two studies in plants (one in soybean seeds and the other in broad bean) revealed that SMFs affected ROS levels probably through different antioxidant enzymes. More studies can be found in Table 6. Undoubtedly, the effects of MFs on the activities of antioxidant enzymes are still inconclusive and the mechanisms that cause these differential changes are also unclear. More in-depth investigations are still necessary, such as MF-induced changes in mitochondrial membrane potential, as well as enzymatic activities in vitro.

## 6. Summary and Future Perspectives

ROS play vital roles in many cellular signaling pathways under both physiological and pathological conditions. Here, we review the effects of MFs on ROS levels, and find that in most cases, MFs (SMFs, ELF-EMFs, and RF-EMRs) could increase ROS levels in multiple types of human, mouse, and rat cells, as well as in various mice and rat tissues. However, a few evidences also showed that MFs reduced or did not change ROS levels. This discrepancy is largely due to the differences in cell types, MF intensity/frequency/exposure time, specific tissues of animal models, or even assay time-point. Further mechanistic studies are essential for us to get a more complete understanding for the effects of MFs on ROS.

## Figures and Tables

**Table 1:** Reactive oxygen species (ROS) changes induced by static MFs (SMFs).

Species	Cell Lines/Organisms	SMF	Exposure Time	ROS Levels	Specific ROS	Refs.
**Human cells**	Human fibrosarcoma cancer cell line (HT1080)	Low level MF (0.2–2 μT, GMF as control, 45–60 μT)	6/12/24 h	Increased *	H_2_O_2_	[26]
	Neuroblastoma cells (SH-SY5Y)	2.2 mT	24 h	Increased		[27]
		31.7–232.0 mT			•O_2_^−^	[28]
	Monocyte tumor cells (U937)	6 mT	2 h		H_2_O_2_	[29]
	Peripheral blood neutrophils	60 mT (S pole)	45 min		H_2_O_2_/HOCl	[30]
	Diploid embryonic lung fibroblast cell (WI-38)	230–250 mT	18 h		H_2_O_2_	[31]
	Leukemia cells (THP-1)	1.2 T	24 h			[32]
	Human-hamster hybrid A(L) cells, mitochondria-deficient rho(0) A(L) cells, and double-strand break (DSB) repair-deficient XRS-5 cells	8.5 T	3 h			[33]
**Mouse cells**	Embryonic stem (ES) cell (CGR8)-derived embryoid bodies and ES cell-derived Flk-1^+^ cardiovascular progenitor cells	0.2–5 mT	1 h/day, 10 days			[34]
	Normal liver cell line (NCTC 1469)	0.4 T	1/24/48/72 h			[35]
	Embryonic Stem Cells	1/10 mT	8 h/day, 17 days			[36]
**Bovine cells**	Bovine pulmonary artery endothelial cells (PAEC)	Low level MF (0.2–2 μT, GMF as control, 45–60 μT)	8/24 h	Increased *		[26]
**Plant**	Soybean seeds	150–200 mT	1 h	Increased	•O_2_^−^/•OH/H_2_O_2_	[37]
**Human cells**	Peripheral blood neutrophils	60 mT	15 min	Decreased	H_2_O_2_/HOCl	[30]
	Bronchial epithelial cells (A549)	389 mT	30 min	Decreased RWPE-induced ROS	H_2_O_2_	[38]
**Mouse cells**	Primary mouse skeletal muscle cell	<3 μT (GMF as control, ~50 μT)	3 days	Decreased *		[39]
**Human cells**	Pancreatic cancer cell line (AsPC-1)	Low level MF (0.2–2 μT, GMF as control, 45–0 μT)	12/24 h	No change	H_2_O_2_	[26]
	Peripheral blood neutrophils	60 mT	30 min		H_2_O_2_/HOCl	[30]
			45 min (N pole)			
	Diploid embryonic lung fibroblast cell (WI-38)	230–250 mT	5 days		H_2_O_2_	[31]
	Lung fibroblasts (MRC-5)	370 mT	1 h/day, for 4 days			[40]
	Bronchial epithelial cells (A549)	389 mT	30 min			[38]
**Bacteria**	*E. coli* and *S. aureus*	100 mT				[41]

Grey color indicates that SMFs increase ROS levels. Blue color indicates that SMFs decrease ROS levels, and green color indicates SMFs do not affect ROS levels. “Increased *” means indirect evidence of SMF-induced ROS increase, because the study showed that H_2_O_2_ decreased after GMF shielding. “Decreased *” means indirect evidence of SMF-induced ROS decrease, because the study showed H_2_O_2_ increased after GMF shielding.

**Table 2:** ROS changes in human cells induced by extremely low frequency electromagnetic fields (ELF-EMFs).

Cell Lines/Animal	ELF-EMF		Exposure Time	ROS Levels	Specific ROS	Refs.
	Frequency	Intensity				
Jurkat cells	7.5 Hz	0.4 T	2 h	Increased	H_2_O_2_	[48]
Leukemia cells (K562)	50 Hz	0.025–0.1 mT	1 h		•O_2_^−^	[49]
		1 mT	3 h			[50]
			24 h			[51]
		5 mT	1 h			[52]
Neuroblastoma cells (SH-SY5Y)		100 μT	24 h (measured at 15 days)		H_2_O_2_	[53]
			24 h (measured at 45 days)			[54]
		1 mT	24/48/72 h		•O_2_^−^/H_2_O_2_	[55]
Amniotic epithelial cells (FL)		0.2 mT	15 min		H_2_O_2_	[56]
		0.4 mT	5/15/30 min		•O_2_^−^/H_2_O_2_	
			15/30 min		•O_2_^−^ in mitochondria	
			30/120 min			[57]
			5/15/30 min		H_2_O_2_	[58]
Keratinocyte cells (NCTC 2544)		0.05/0.1 mT	1/2 h		H_2_O_2_/HOCl	[59]
Umbilical cord blood monocytes		1 mT	5/15/30/45 min			[60]
Umbilical cord blood monocytes and acute monocytic leukaemia cell (Mono Mac 6)			45 min		•O_2_^−^	[61]
Bone marrow mesenchymal stem cells (hBM-MSCs)			90 min		H_2_O_2_	[62]
			12 days			[63]
Osteosarcoma cells (MG-63, MNNG-GOS C1)			1/2/3 h			[64]
Prostate carcinoma cells (DU145, PC3, and LNCaP)	60 Hz		6/24/48/72 h			[65]
Breast carcinoma cells (T47D)	217 Hz	0.1 mT	72 h			[66]
Renal proximal tubular cells (HK-2)	10 Hz	1 mT	N/A	Decreased	H_2_O_2_	[67]
Microglial cells (HMO6)	50 Hz		4 h	Decreased oxygen-glucose deprivation-induced ROS		[68]
Neuroblastoma cells (SK-N-BE(2))	75 Hz	2 mT	15 min/day, 3 days	Decreased H_2_O_2_-induced ROS		[69]
Neuroblastoma cells (SH-SY5Y)		1.5 ± 0.2 mT	24/48 h	Decreased hypoxia-induced ROS		[70]
	75 ± 2 Hz	2 ± 0.2 mT	10 min, 4 times/week	Decreased H_2_O_2_-induced ROS		[71]
Keratinocytes and dermal fibroblasts	100 Hz	<40 μT	24 min twice daily, 30 days	Decreased		[72]

Grey color indicates that ELF-EMFs increase ROS levels. Blue color indicates that ELF-EMFs decrease ROS levels. “N/A” means that we did not find relevant information of exposure time.

**Table 3:** ROS levels were not changed by ELF-EMFs in some human cells.

Cell Lines/Animal	ELF-EMF		Exposure Time	ROS Levels	Specific ROS	Refs.
	Frequency	Intensity				
Jurkat cells	7.5 Hz	0.4 T	1/3 h	No change	H_2_O_2_	[48]
Renal proximal tubular cell	10 Hz	0.01/0.1 mT	N/A			[67]
Keratinocyte cells (NCTC 2544)	50 Hz	0.025/0.15/0.2 mT	1 h		H_2_O_2_/HOCl	[59]
		0.05/0.1 mT	4 h			
Neuroblastoma cells (SH-SY5Y)		100 μT	24 h (measured at 8/15/30 days)		H_2_O_2_	[53]
						[54]
Amniotic epithelial cells (FL)		0.1 mT	15 min			[56]
		0.4 mT	5 min		•O_2_^−^ in mitochondria	
			60 min		•O_2_^−^/ H_2_O_2_/•O_2_^−^ in mitochondria	
					•O_2_^−^ in mitochondria	[57]
					H_2_O_2_	[58]
Jurkat cells		1 mT	1 h (5 min on/10 min off)			[73]
Prostate carcinoma cells (DU145, PC3, and LNCaP)	60 Hz	1 mT	3 h			[65]
Normal breast epithelial cells (MCF10A)			4 h			[74]
Lung fibroblast (IMR90) and cervical carcinoma (HeLa) cells		7 mT	30/60 min			[75]
Neuroblastoma cells (SK-N-BE(2))	75 Hz	2 mT	15 min/day, 3 d	No change in cells without H_2_O_2_		[69]
Neuroblastoma cells (SH-SY5Y)	75 ± 2 Hz	2 ± 0.2 mT	10 min, 4 times/week			[71]
Renal proximal tubular cells (HK-2)	50/100 Hz	1 mT	N/A	No change		[67]
Neutrophils	180–195 Hz	10/40/60 μT	N/A		H_2_O_2_/HOCl	[76]
Breast carcinoma cells (T47D)	100 Hz	0.1 mT	24/48/72 h		H_2_O_2_	[66]
	217 Hz		24/48 h			

“N/A” means that we did not find relevant information of exposure time.

**Table 4:** ROS changes in mice and rats induced by ELF-EMFs.

Species	Cell Lines/Animal	ELF-EMF		Exposure Time	ROS Levels	Specific ROS	Refs.
		Frequency	Intensity				
**Mouse cells**	Primary mouse T cells from female C57BL/6 mice	7.5 Hz	0.4 T	2 h	Increased	H_2_O_2_	[48]
	Undifferentiated C2C12 cells (myoblasts) and terminally differentiated myotubes	50 Hz	1 mT	5/30 min			[77]
	Squamous cell carcinoma cells (AT478)			16 min			[78]
	Bone marrow-derived (MBM) macrophages			45 min		H_2_O_2_/HOCl	[24]
	Embryonic fibroblasts		2 mT	2/6 h		H_2_O_2_	[79]
	Embryonic stem (ES) cell-derived embryoid bodies		1/10 mT	8 h/day, 7 days			[22]
**Rat cells**	Undifferentiated pheochromocytoma-derived cells (PC12)		1 mT	30 min			[80]
	Rat immortalized fibroblasts (Rat1)			3/24 h			[81]
	Primary hippocampal neurons		8 mT	90 min			[3]
	Rat peritoneal neutrophils	60 Hz	0.1 mT	5 days			[82]
**Mouse**	Hippocampus mitochondria of male ICR mice	50 Hz	8 mT	4 h/day, 28 days			[83]
**Rat**	Male Wistar rats	40 Hz	7 mT	60 min/day, 14 days			[84]
	Hippocampus/cerebellum of male Wistar rats	50 Hz	50 μT	90 days			[85]
	Hippocampus/cerebellum/cortex of male Wistar rats		100 μT				
**Mouse cells**	Squamous cell carcinoma cells (AT478)	50 Hz	1 mT	16 min	Decreased cisplatin-induced ROS	H_2_O_2_	[78]
	Mouse microglial cells (N9)	75 Hz	1.5 ± 0.2 mT	24/48 h	Decreased hypoxia-induced ROS		[70]
**Rat cells**	Primary cardiomyocytes from neonatal Sprague-Dawley (SD) rat hearts	15 Hz	4.5 mT	3 h	Decreased hypoxia/reoxygenation (H/R)-induced ROS	•O_2_^−^	[86]
	Pheochromocytoma cells (PC12)	75 Hz	1.5 ± 0.2 mT	24/48 h	Decreased hypoxia-induced ROS	H_2_O_2_	[70]
**Mouse cells**	Primary mouse T cells from female C57BL/6 mice	7.5 Hz	0.4 T	1/3 h	No change	H_2_O_2_	[48]
	Undifferentiated C2C12 cells (myoblasts) and terminally differentiated myotubes	50 Hz	0.1 mT	5/30 min			[77]
	Bone marrow-derived (MBM) macrophages		1 mT	5/15/30 min		H_2_O_2_/HOCl	[24]
	Embryonic fibroblasts		2 mT	0.5/12/24 h		H_2_O_2_	[79]
	Undifferentiated PC12 cells		0.1 mT	30 min			[80]
	Differentiated PC12 cells		0.1/1 mT				
**Rat cells**	Rat-cortical neurons (from SD rat embryos)			7 d			[87]
	Naive/chemically stressed PC12		1 mT	30 min/48 h			[88]
**Rat**	Male Wistar rats	40 Hz	7 mT	30 min/day, 14 days			[84]
	Male SD rats			30/60 min/day, 10 days			[89]
	Cortex of male Wistar rats	50 Hz	50 μT	90 days			[85]

Grey color indicates that ELF-EMFs increase ROS levels. Blue color indicates that ELF-EMFs decrease ROS levels, and green color indicates ELF-EMFs do not affect ROS levels. “N/A” means that we did not find relevant information of exposure time.

**Table 5:** ROS changes induced by radio frequency electromagnetic radiations (RF-EMRs).

Species	Cell Lines/Organisms	RF-EMR		ROS Levels	Specific ROS	Refs.
		Frequency	Time			
**Human cells**	Ejaculated semen	870 MHz	60 min	Increased	H_2_O_2_	[91]
	Spermatozoa	1.8 GHz	16 h		•O_2_^−^	[92]
	Neuroblastoma cells (SH-SY5Y)	872 MHz	1 h	Increased menadione-induced ROS	H_2_O_2_	[93]
	Peripheral blood mononuclear cell	900 MHz	1/2/4/6/8 h	Increased		[94]
	HEK293T-harbouring the firefly luciferase gene	940 MHz	5/15/30/45 min			[95]
	Lens epithelial cells	1.8 GHz (3/4 W/kg)	24 h			[96]
**Rat cells**	Pulmonary arterial smooth muscle cells (rPASMC)	7 MHz	2/3 days			[97]
	Primary neocortical astroglial cell	900 MHz CW modulated in 50 Hz AM	20 min			[98]
	Male Wistar rat semen	900 MHz	2 h/day, 35 days		Total ROS	[99]
		10 GHz	2 h/day, 45 days			[100]
**Rat**	Serum of male Wistar rats	900 MHz				[101]
**Drosophila**	Male/female drosophila bodies	1.88–1.90 GHz	6/24/96 h		H_2_O_2_	[102]
	Ovaries of female drosophila		0.5/1/6/24/96 h			
**Rat cells**	Pulmonary arterial smooth muscle cells (rPASMC)	7 MHz	3 days	Decreased	•O_2_^−^	[97]
**Human cells**	Neuroblastoma cells (SH-SY5Y)	872 MHz	1 h	No change	H_2_O_2_	[93,103]
	Primary human thyroid cells	900/895 MHz	3/16 h (900 MHz)/65 h (895 MHz)		Total ROS	[104]
	Primary monocytes and lymphocytes	1800 MHz (CW/intermittent)	30/45 min		H_2_O_2_/HOCl	[105]
	Lens epithelial cells	1.8 GHz (1/2 W/kg)	24 h		H_2_O_2_	[96]
**Mouse cells**	Murine fibrosarcoma cells (L929)	900 MHz (CW or GSM)	10/30 min			[106]
**Rat cells**	Primary neocortical astroglial celll	900 MHz CW modulated in 50 Hz AM	5/10 min			[98]
		900 MHz CW	5/10/20 min			
	Lymphocytes (male albino Wistar rats)	930 MHz	5/15 min			[107]
**Drosophila**	Male/female drosophila bodies	1.88–1.90 GHz	0.5/1 h			[102]
***C. elegans***	Caenorhabditis elegans	DECT/WI-FI/GSM	Dependent on strains and devices			[108]

Grey color indicates that RF-EMRs increase ROS levels. Blue color indicates that RF-EMRs decrease ROS levels, and green color indicates RF-EMRs do not affect ROS levels.

**Table 6:** The magnetic field (MF)-induced activities changes of antioxidant enzymes.

MF Types	Species	Cell Lines/Organisms	MF Exposure		Antioxidant Enzymes	Refs.
			Conditions	Time		
**SMFs**	Mouse	Cochlea tissue of C57BL/6 mice	5 mT	1/3/5/7/14 days (8 h first day, 2 h/day for the rest)	CAT and SOD activities significantly increased only after 3 days exposure, but not others	[44]
	Plant	Soybean seeds	150–200 mT	1 h	SOD activity was reduced	[37]
		Broad bean (*Vicia faba* L.)	15 mT	8 h/day, 8 days	SOD increased, CAT decreased, indirectly suggest ROS accumulation	[111]
**ELF-EMFs**	Mouse cells	Preadipocyte cell (3T3-L1)	180–195 Hz, 120 μT	36 min/day, 2 days	SOD decreased, CAT increased, GSH-Px and GSSG-Rd with no change after 24 h; but SOD, CAT, and GSH-Px significantly decreased, GSSG-Rd with no change after 48 h	[112]
	Mouse	Balb/c mouse brain	60 Hz, 1.2 mT	3 h	SOD increased	[113]
**RF-EMRs**	Rat cells	Male Wistar rats sperm	50 GHz	2 h/day, 45 days	CAT significantly increased, SOD and GSH-Px significantly decreased	[114]
	Rat	Female Wistar rats	900 MHz	1 h/day, 7 days	No change (SOD and GSH-Px decreased non-significantly)	[115]
		Male SD rats		30 min/day, 3 months	No change (SOD, CAT and GSH-Px decreased marginally)	[116]
		Brain of male Wistar rats		2 h/day, 45 days	SOD and GSH-Px decreased, CAT increased	[101]
	Rabbit	Male albino rabbits		30 min/day, 7 days	Serum SOD activity increased	[117]

Blue color indicates SMFs, grey color indicates ELF-EMFs, and green color indicates RF-EMRs.
